# The Artificial Feeding of Infants

**Published:** 1892-09

**Authors:** Charles S. Shaw

**Affiliations:** Pittsburg, Pa.


### THE ARTIFICIAL FEEDING OF INFANTS.

BY CHARLES S. SHAW, M. D.,
PITTSBURG, PA.

The proportion of artificially fed infants to those suckled by the mother, or wet nurse, in large cities, is variously estimated to be from ten to twenty per cent. As these estimates are based solely upon individual observation they necessarily differ widely because of the various conditions of environment that determine the method of feeding. Broadly, it may be said, that in the higher social scale the infant is more likely to be deprived of its natural nourishment. My own experience would place the proportion of artificially fed infants at not less than ten per cent. This refers only to infants who are fed exclusively in this manner ; the proportion would be much larger if it included those in whom, because of a deficiency in the quality or quantity of the mother's milk, some artificial adjuvant is necessary. The proper feeding of infants artificially is a subject that may well engage the attention of medical men, for it may be safely asserted that the number of children properly so fed is, unhappily, very small. It is needless to refer to the disasters that attend the bottle-fed baby; they are too familiar to demand anything more than a passing notice. The statement of Holt may be quoted, however, as a general index of infant mortality under these conditions. Of 1,943 cases of fatal diarrhœal disease, ninety-seven per cent. were artificially fed.

With the maternal method of feeding constantly before us as a model, it would seem that a sufficient substitute ought not to be difficult to obtain, but in spite of the apparent simplicity of the problem it has not yet been entirely solved, and until very recently hardly any progress whatever had been made towards its solution. There is, however, a vast improvement in the artificial feeding of infants in the last two years; and the improvement is due, like most recent advances in medicine, to a recognition of the all-pervading influence of bacterial life. The investigations of the bacteriologist, which have so enlarged the power and scope of surgery, which promise so much in the treatment of disease, have also had a most salutary effect upon the subject under consideration.

In dealing with this question of artificial feeding, it is evident that we should endeavor to approximate the maternal and natural method as closely as possible, in the composition of the food, and the quantity, the frequency of feeding and the method of administration.

Practically, we have but one substitute for the mother's milk, that is the milk of the cow. Goat's milk, asses' milk and mare's milk have been suggested and recommended, but it is evident that their usefulness is very restricted. The legions of prepared foods in the market may be dismissed without notice. They are all defective in composition and at most can be used only as adjuvants to a diet whose basis must be milk. The milk at the command of the consumer in a large city is that which we must use. Of course it goes without saying, that the fresher and purer the milk the better, but with the modern method of treatment, the milk in the hands of the dealer is sufficient, so that the care and expense with which the anxious parent used to seek out peculiar excellences in the milk for the child are now largely removed. The endeavor is to make a food of this milk that will resemble the mother's milk as closely as possible.

The average of the analyses of human milk, taken from Rotch's article in the Cyclopedia of Diseases of Children is: water, 87-89 parts; solids, 12-13 parts. These solids are: fat, 4 parts; albuminoids, 1 part; milk sugar, 7 parts; ash, 2 parts. Cow's milk shows, by the same authority, practically the same proportion of water and solids, but the solids are: fat, 4 parts; albuminoids, 4 parts; milk sugar, 4 parts; ash, 7 parts. Here we see the same amount of fat in each, but the cow's milk contains four times as much albuminoids, a little more than one-half as much sugar milk and more than three times as much ash. Evidently if we dilute the cow's milk with water till the albuminoids are the same in each, we will have only one-fourth as much fat, a little more than half as much sugar, while the ash will be nearly the same. Then by the addition of fat and milk sugar in the proper quantity to the diluted cow's milk, we make a mixture very closely resembling the mother's milk. But the cow's milk, as we find it in the market, is acid, while woman's milk is invariably alkaline. To remedy this we add lime water to the mixture, and the result is a compound that seems to be almost identical, chemically, with human milk.

For the preparation of 8 oz. of the mixture, as given by Rotch, take water, 3 oz.; cream 2 oz.; milk, 1 oz.; lime water, one-fourth strength, 2 oz.; milk sugar, 3⅜ drams. One of the essential differences between cow's milk and woman's milk, that is removed by this dilution, is the character of the curd. Undiluted cow's milk coagulates in hard and large curds, while the curd in woman's milk is very light and fine. The dilution of cow's milk, with four parts of water, reduces the curd to about the same as that of woman's milk. This mixture resembles closely the old Meigs' mixture that was so popular in Philadelphia a generation ago. In composition, appearance, taste and reaction it is almost a perfect imitation of human milk, but there is still a most important difference—the mother's milk is sterile; the mixture is not. It is the sterilization of the artificial food that is the great advance in modern infant feeding. The experience of the nursery long ago taught that boiling the milk made it "keep better," and rendered it more acceptable to the infant's stomach. Reason would suggest that raw milk is the more natural, and therefore the better food for the child; and Raudnitz has demonstrated that boiling the milk lessens its nutritive value, but in spite of these objections the boiled milk was preferred. The reason for this preference is that boiled milk is sterilized, and sterilization is of more importance than comparative nutritive value or digestibility. The reason for the well-merited popularity of condensed milk may be found in the same fact. Condensed milk, though a very imperfect food, chemically, is more or less sterile, and this compensates for all its shortcomings. Now, if we sterilize the mixture above described, we have counterfeited the mother's milk in every essential particular, and have, if not the ideal food, at least a very satisfactory one, and the best at present attainable.

This is done by heat. The most convenient way is by subjecting the mixture to a steam bath for twenty or thirty minutes, though any other method of thorough heating will answer. The only requisite is that the mixture shall be heated to a temperature of at least 212°, and kept at that heat for twenty minutes or more. The vessel containing the mixture may be placed in a water bath and heated, or a steam sterilizer may be improvised with the kitchen utensil known as a colander, and a pot of boiling water. It is, however, better both for efficiency and convenience to use some apparatus designed for the purpose. Various forms of sterilizers are on the market, and they may be equally good, but my experience has been limited to that known as the Arnold Steam Sterilizer, with which you may be familiar. It consists of an evaporating pan surmounted by a steam chamber, which holds a rack containing a number of 7 oz. graduated bottles. It is made of tin and is inexpensive. In using this apparatus the child's food for the day is prepared when the milk arrives in the morning. When making the mixture the lime water should not be added until after heating. Otherwise the food will have a straw yellow color, and the taste will not be as pleasant, though the nutritive value and wholesomeness are not impaired. Sufficient of the mixture, without the lime water, for each nursing is put into each bottle, and the whole sterilized by thirty minutes steaming. The proper quantity of lime-water is then added to each bottle, the neck of each is stopped by a plug of clean cotton, and as required they are used. In this apparatus the sterilizing bottles are used as nursing bottles, and very excellent ones they make. The plug of cotton is removed, the rubber nipple adjusted, and the mixture heated to about 100° in warm water. Any food remaining in the bottle after the infant has satisfied its appetite, is thrown out. This use of the bottle is not only a great convenience, but it also removes all necessity for disturbing or handling the mixture after sterilizing, and renders subsequent contamination of the food improbable.

The quantity of this sterilized mixture to be given to the child at each feeding depends of course on the size and vigor of the infant, and its digestive capacity. Biedert's law is two and a half ounces of the food to every pound weight of the child in 24 hours. During the first three months the babe may be fed every two hours during the day, and every four hours during the night. This would make ten feedings in the twenty-four hours, and, allowing one and a half or two ounces at each feeding, would amount to a pint or a little more of the mixture. There is always more likelihood of over-feeding than under-feeding. As the child grows the quantity of the food should be increased, and the intervals between the feeding lengthened. At six months the child may be taking four or four and a half ounces of food at each feeding, and a total of two and a half pints in the twenty-four hours.

If the sterilizing bottle is not used in feeding the child, the best substitute is the ordinary nursing bottle, or a common prescription vial of four or six-ounce capacity. This bottle is surmounted by the common rubber nipple or teat. As found in the shops the perforations in this rubber nipple are frequently too minute, and should be enlarged. In the selection of the nipple care should be taken that it is neither too hard and unyielding, a common fault with those made of white rubber, nor so soft and flaccid that it will collapse, as some of the black rubber and red rubber ones do. The worst apparatus is probably that arrangement with the long rubber tube, so popular with lazy and ignorant nurses, and to be had at all drug stores. It is a device worthy of the invention of a Herod, and its sale should be prohibited by law. When feeding, the infant should always be held in the nurse's arms in a semi-upright position, and the bottle should be held by the nurse, and the flow of food regulated by tilting the bottle. There are several designs in nursing bottles for the admission of air through a valve, but none of them are satisfactory, and all are objectionable, because of the difficulty of keeping them clean. The nursing bottle and teat should be thoroughly cleansed after each feeding, and when not in use should be kept in a weak solution of soda or borax.

Up until the tenth month the best results will follow the use of this sterilized mixture to the exclusion of all other food; but with the advent of dentition comes the abilty to digest and assimilate other forms of food, and these may be judiciously added, keeping the original diet as the basis of nourishment. The common household preparation of flour, arrowroot, corn starch, etc., may come gradually into use, supplemented by meat juices and well cooked fruits.

